# Elevator-type mechanisms of membrane transport

**DOI:** 10.1042/BST20200290

**Published:** 2020-05-05

**Authors:** Alisa A. Garaeva, Dirk J. Slotboom

**Affiliations:** 1 Groningen Biomolecular Sciences and Biotechnology Institute, Membrane Enzymology, University of Groningen, The Netherlands; 2 Zernike Institute for Advanced Materials, University of Groningen, The Netherlands

**Keywords:** membrane proteins, protein structure, transport

## Abstract

Membrane transporters are integral membrane proteins that mediate the passage of solutes across lipid bilayers. These proteins undergo conformational transitions between outward- and inward-facing states, which lead to alternating access of the substrate-binding site to the aqueous environment on either side of the membrane. Dozens of different transporter families have evolved, providing a wide variety of structural solutions to achieve alternating access. A sub-set of structurally diverse transporters operate by mechanisms that are collectively named ‘elevator-type’. These transporters have one common characteristic: they contain a distinct protein domain that slides across the membrane as a rigid body, and in doing so it ‘drags” the transported substrate along. Analysis of the global conformational changes that take place in membrane transporters using elevator-type mechanisms reveals that elevator-type movements can be achieved in more than one way. Molecular dynamics simulations and experimental data help to understand how lipid bilayer properties may affect elevator movements and vice versa.

## Introduction: moving barriers and elevators

**Figure 1.**
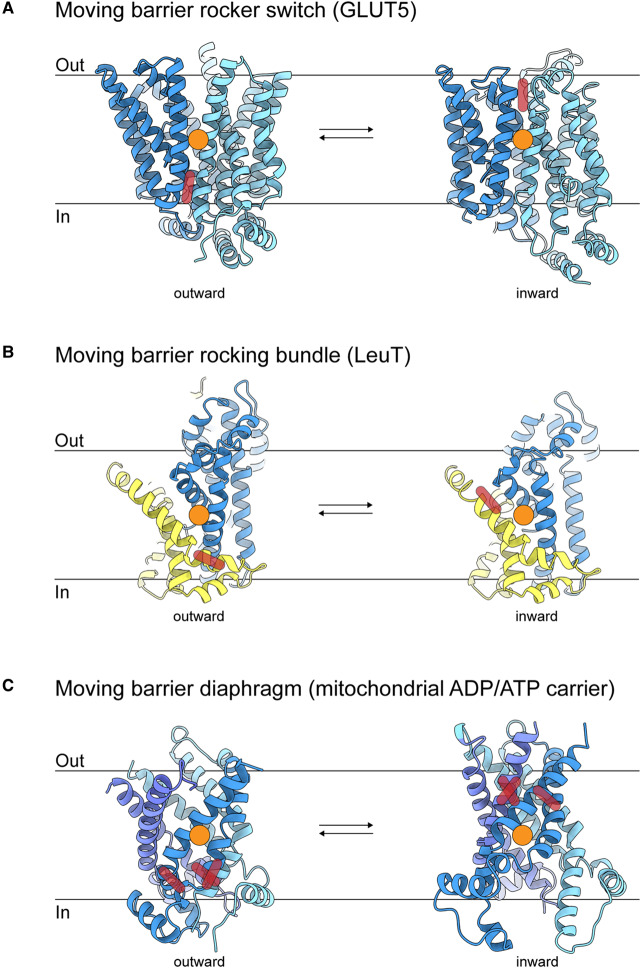
Non-elevator type transporters. (**a**) moving barrier, rocker switch, exemplified by the fructose transporter GLUT5 with two protein domains (blue shades) rotating around substrate-binding site (orange circle) changing the barrier position (red bars) (PDB IDs for outward and inward states: 4YBQ and 4YB9).(**b**) moving barrier, rocking bundle, exemplified by the leucine transporter LeuT with transport domain (blue) moving relative to the scaffold domain (yellow). The substrate-binding site does not change its position relative to the membrane plane during the transition from outward to the inward state, but the barrier (red bar) does change (PDB IDs: 3TT1 and 3TT3). (**c**) the mitochondrial ADP/ATP carrier represents the moving-barrier, diaphragm mechanism, where three protein domains (blue shades) rotate around substrate-binding site changing the barrier position, indicated by the red bars (PDB IDs: 6GCI and 4C9H).

Structural studies of membrane transporters from diverse protein families have revealed that alternating access may by achieved in many ways (reviewed recently [1]). The so-called “moving barrier” mechanism is a frequently used solution (Figure 1). Proteins operating by this mechanism bind the transported substrate in a deep cavity, which is accessible to the aqueous environment from one side of the membrane only. A conformational change then closes off the access path to the binding site (gate closure), and opens up a new path to the other side of the membrane (gate opening). Moving barrier transporters thus work with two separate gates. Synchronization of opening and closing of the two gates is crucial: intermediate occluded states with both gates closed may be visited, but states with both gates open are prohibited. During the conformational transitions in the protein, the substrate remains bound at roughly the same position relative to the bilayer plane, until the conformational switching has been completed and a route to the aqueous solution on the opposite side of the membrane has opened. In many cases, the substrate-binding site is located halfway through the bilayer between two proteins domains that move around the substrate when switching between inward- and outward-facing states. The transport protein thus serves as a “moving barrier’. Prominent examples of proteins using a moving barrier mechanism include members of the major facilitator superfamily, in which two homologous protein domains swivel around the substrate as a rocker switch [2,3] (Figure 1a); the LeuT-fold proteins in which one protein domain moves as a rocking bundle relative to a fixed second (non-homologous) domain [4] (Figure 1b); and mitochondrial carriers, where three homologous domains pivot around the substrate in a concerted way as a diaphragm [5] (Figure 1c).

**Figure 2.**
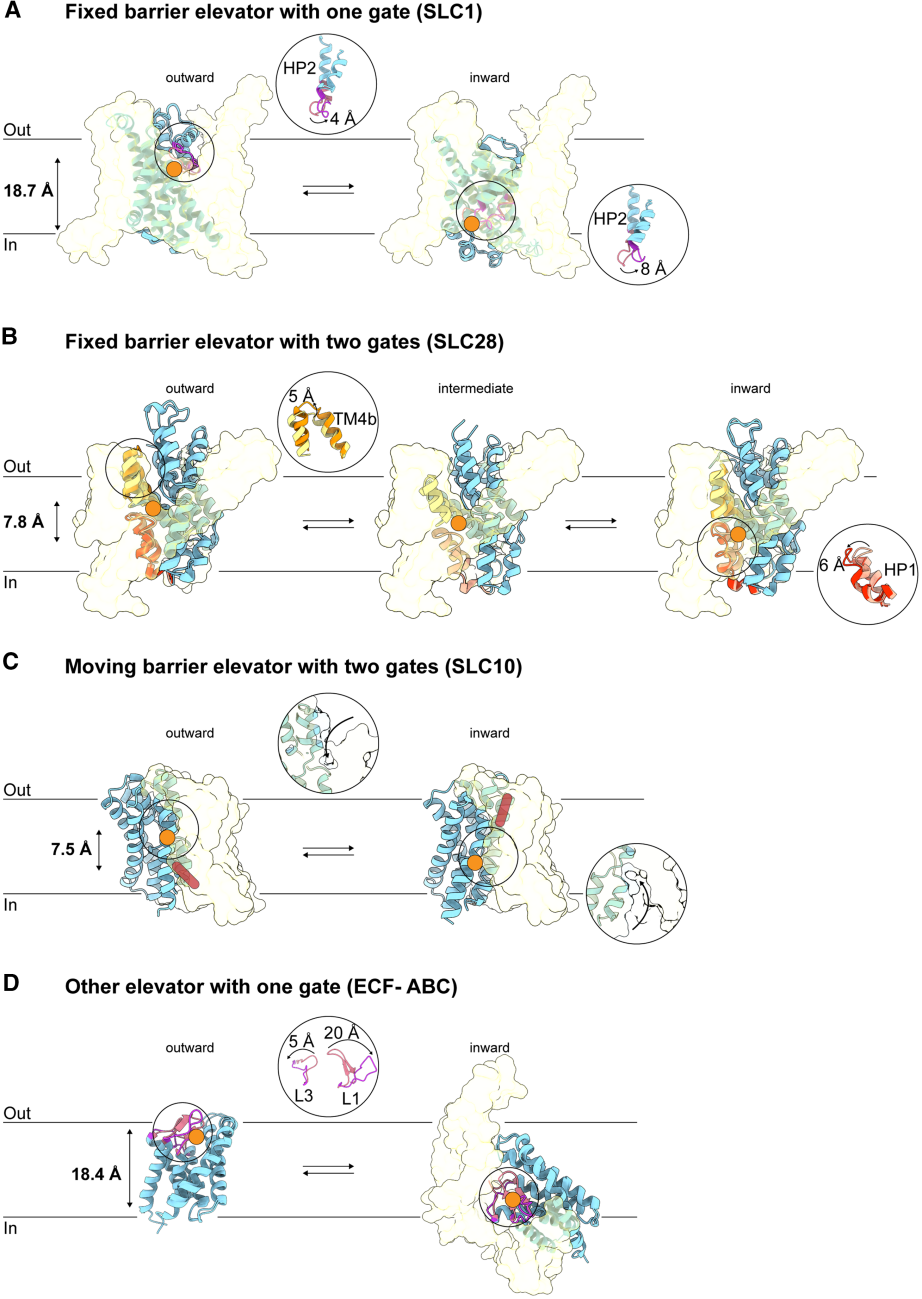
One- and two-gate elevators. (**a**) fixed barrier elevator with one gate. Neutral amino acid transporter ASCT2 (SLC1 family) (transport domain as blue ribbon; scaffold domain as yellow transparent surface) uses helical hairpin HP2 as a gate in both the outward state (it moves by 4 Å form the light pink closed (PDB ID: 6MPB) to the bright pink open conformation (PDB ID: 6MP6)) and in the inward state (8 Å movement from closed (PDB ID: 6GCT) to open position (PDB ID: 6RVX)). ASCT2 translocates substrate (orange circle) relative to the membrane plane during transport (distances are indicated on the left), keeping the same contact (barrier) with the stable scaffold domain. (**b**) fixed barrier elevator with two gates. Concentrative nucleoside transporter CNT (SLC28 family) uses TM4b as an extracellular gate (5 Å movement from closed yellow (PDB ID: 5U9W, chain C) to open orange state (PDB ID: 5L2A, chain C)) and HP1 as an intracellular gate (6 Å movement from light pink closed (PDB ID: 5L26, chain A) to red open state (PDB ID: 5L27, chain A)). CNT is the only elevator transporter, for which multiple intermediate conformations have been resolved structurally, one of which is shown (PDB ID: 5L24, chain C). (**c**) moving barrier elevator with two gates. The bile acid transporter ASBT (SCL10 family) provides access to the binding site (indicated by arrows within the circle) using bundle movements of the transport domain (PDB ID: 4N7X and 3ZUX), during which barrier (red bar) is changing. (**d**) other elevator with one gate. Energy coupling factor folate transporter ECF-FolT (ECF-type (type III) ABC importer) has loop 1 (L1) and loop 3 (L3) in the S-component (blue ribbon) that provide access to the substrate-binding site from the extracellular (PDB ID: 5D0Y) and the intracellular side (PDB ID: 5JSZ). The EcfT subunit is in yellow transparent surface, and the ATPase subunits are omitted for clarity.

The elevator-type transport mechanism offers an alternative solution to achieve alternating access [1]. Proteins using this mechanism consist of a moving and fixed domain (often termed “transport” and “scaffold” domain, respectively). Switching between outward- and inward-facing states involves the sliding of the entire transport domain through the bilayer as a rigid body. In contrast with proteins using a moving-barrier mechanism, the substrate-binding site translocates some distance across the bilayer during transport along with the transport domain (Figure 2). Because of the displacement of the substrate the elevator mechanism has been described as “moving carrier’. Alternatively, the name “fixed barrier mechanism” has been proposed [1], but as we will discuss below, some elevator proteins may not have a fixed barrier. Therefore, we prefer the names “elevator-type” or “moving carrier” mechanism. It is noteworthy that the classification of a transporter mechanism as “moving barrier” or “moving carrier” is based solely on the structural changes that take place in the proteins during transport, and that it does not have predictive value for the transporter's substrate specificity, coupling ion specificity (in secondary active transporters), or for the kinetic mechanism.

The first elevator-type mechanism was described in 2009 for the aspartate transporter Glt_Ph_ [6], a member of the glutamate transporter or SLC1 (Solute Carrier 1) family, but the name “elevator” was not used until 2011 [7]. In recent years, elevator-type mechanisms have been proposed for numerous other proteins (Table 1). Many of the proteins shown in Table 1 are sodium-coupled secondary active transporters, but a sub-set of ATP-binding cassette (ABC) transporters, phosphotransferase system (PTS) transporters and unclassified transport proteins also appear to use elevator-type mechanisms. The abundant representation of secondary active transporters in Table 1 may simply be a reflection of the large number of families of secondary transporters that have evolved [8]. In this review, we focus on the global structural changes that take place in elevator-type membrane transporters. We do not discuss the kinetics of switching between outward- and inward-facing states, which may depend on the occupancy of the solute-binding site, or binding of compounds to allosteric sites, such as co-transported ion(s) in secondary active transporters, or nucleotides in ATP-binding cassette (ABC) transporters. For details of the intricate mechanisms of coupling of transport to co-ion translocation or ATP hydrolysis we refer to recent reviews [9–12].

**Table 1.** Available structures and characteristics of the transporters with proposed elevator-like transport mechanism

Protein	Outward-facing conformation (PDB accession code)	Inward-facing conformation (PDB accession code)	Intermediate conformation (PDB accession code)	Oligomeric state	Protein family	Total substrate-binding site displacement (Å)^1^	Vertical displacement (Å)^1^	Number of helical hairpins	Substrate binding site location	Type of elevator	Method of structure determination	Topology of inverted repeats
ASCT2	6mp6[27] 6mpb[27]	6gct[29] 6rvx[28] 6rvy[28]	-	Trimer	SLC1	20.2	18.7	2	within the transport domain	fixed barrier with one gate	cryo-EM,	present
Glt_Tk_	4ky0[62] 5dwy[63] 5e9s[63] 6r7r[64] 6xwn[30]	6xwr[30] 6xwo[30] 6xwp[30] 6xwn[30]	6xwr[30] 6xwo[30] 6xwp[30] 6xwq[30]	Trimer	SLC1	23.7	21.2	2	within the transport domain	fixed barrier with one gate	X-ray, cryo-EM	present
Glt_Ph_	1xfh[65] 2nww[66] 2nwl[66] 2nwx[66] 4izm[67] 4oye[68] 4oyf[68] 5cfy[68] 6ctf[58] 6bat[69] 6bau[69] 6bav[69] 6bmi[69]	3kbc[6] 3v8f[70] 4p6h[68] 4p19[68] 4p1a[68] 4p3j[68] 4x2s[54]	3v8g[70]	Trimer	SLC1	21	18	2	within the transport domain	fixed barrier with one gate	X-ray	present
EAAT1	5llm[47] 5llu[47] 5lm4[47] 5mju[47]	-	-	Trimer	SCL1			2	within the transport domain	fixed barrier with one gate	X-ray	present
CNT_NW_	5l2a[36] 5l2b[36]	5l26[36]	5l27[36] 5l24[36] 5u9w[36]	Trimer	SLC28	10.9	7.8	2	at the interface	fixed barrier with two gates	X-ray	present
vcCNT	-	3tij[71] 4pb1[72] 4pb2[72] 4pd5[72] 4pd6[72] 4pd7[72] 4pd8[72] 4pd9[72] 4pda[72]	-	Trimer	SLC28			2	at the interface	fixed barrier with two gates	X-ray	present
ASBT_NM_	-	3zux[73] 3zuy[73]	-	monomer	SLC10	8.7	7.5	0	at the interface	moving barrier with two gates	X-ray	present
ASBT_Yf_	4n7w[44] 4n7x[44]	-	-	monomer	SLC10	8.7	7.5	0	at the interface	moving barrier with two gates	X-ray	present
Bor1	-	5l25[74] 5sv9[75]	-	dimer	SLC4			0	at the interface		X-ray, electron crystallography of 2D crystals	present
AE1	4yzf[39]	comp.model[15]	-	dimer	SLC4	11[15]	8[15]	0	at the interface		X-ray, modelling	present
UraA	-	3qe7[41] 5xls[40]	-	dimer	SLC23			0	at the interface		X-ray	present
UapA	-	5i6c[76]	-	dimer	SLC23			0	at the interface		X-ray	present
SLC26Dg	-	5da0[77]	-	dimer	SLC26	6[42]		0	at the interface		X-ray	present
BicA	-	6ki1[43] 6ki2[43]	-	dimer	SLC26		6[43]	0	at the interface		X-ray, cryo-EM	present
MtrF	-	4r1i[78]	-	dimer	AbgT			2	at the interface		X-ray	present
YdaH	-	4r0c[79]	-	dimer	AbgT			2	at the interface		X-ray	present
KpCitS	5x9r[80] 5xas[80]	4bpq[81] 5xat[80] 5xar[80] 5xas[80]	-	dimer	2HCT	14.6	13.9	2	at the interface	fixed barrier	X-ray, electron crystallography of 2D crystals	present
SeCitS	5a1s[38]	5a1s[38]	-	dimer	2HCT	17.3	15.2	2	at the interface	fixed barrier	X-ray	present
VcINDY	comp.model[14]	4f35[82]	-	dimer	DASS	15[14]		2	at the interface		X-ray, modelling	present
EcNhaA	-	1zcd[83] 4au5[84] 4atv[84] 3fi1[85]	-	dimer	Na^+^/H^+^ antiporters	10[86]		0	at the interface	moving barrier with two gates	X-ray, electron crystallography of 2D crystals	present
TtNapA	4bwz[86] 5bz3[48]	5bz2[48]	-	dimer	Na^+^/H^+^ antiporters	9.6	8.6	0	at the interface	moving barrier with two gates	X-ray	present
MjNhaP1	-	4czb[87]	-	dimer	Na^+^/H^+^ antiporters			0	at the interface		electron crystallography of 2D crystals	present
PaNhaP	-	4cz8[88] 4cz9[88] 4cza[88]	-	dimer	Na^+^/H^+^ antiporters			0	at the interface		X-ray	present
bcMalT	5iws[32]	6bvg[33]	-	dimer	PTS system	11.5	9	2	at the interface	fixed barrier	X-ray	present
bcChbC	-	3qnq[34]	-	dimer	PTS system			2	at the interface		X-ray	absent
ecUlaA	4rp8[89] 4rp9[89]	-	-	dimer	PTS system	18.8	16.6	4	at the interface	moving barrier	X-ray	present
pmUlaA	-	5zov[90]	-	dimer	PTS system	18.8	16.6	4	at the interface	moving barrier	X-ray	present
TtCcdA	5vkv[17]	comp.model[17]	-	monomer	LysE	12[17]		0	at the interface	moving barrier	NMR, modelling	present
ECF transporters	4m58[91] 4m5c[91] 4m5b[91]	5x3x[92] 5x41[92]	-	Protein complex	Group I ECF ABC			0	within the transport domain	one-gate elevator	X-ray	absent
ECF transporters	5d0y[35] 3p5n[93] 3rlb[94] 4dve[95] 5kbw[96] 5kc0[96] 5kc4[96] 4mes[97] 4mhw[97] 4muu[97] 4pop[97] 4pov[97] 4n4d[97] 4z7f[98] 6ffv[99]	5jsz[35] 5d3m[35] 6fnp[100] 4rfs[101] 4huq[102] 4hzu[103]	-	protein complex	Group II ECF ABC	22.1	18.4	0	within the transport domain	one-gate elevator	X-ray	absent

1 See text for definitions and abbreviations.

## Common characteristics of elevator-type transporters

**Figure 3.**
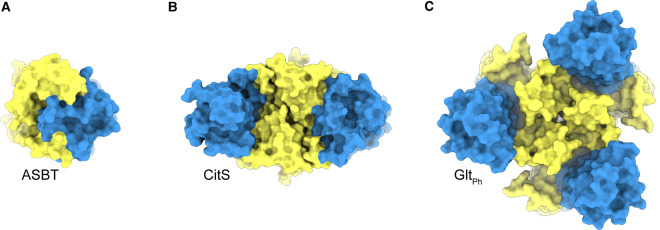
Oligomeric state of elevator transporters. (**a**) monomeric bile acid transporter ASBT (PDB ID: 3ZUX), (**b**) dimeric citrate transporter SeCitS (PDB ID: 5A1S) and (**c**) trimeric glutamate transporter Glt_Ph_ (PDB ID: 2NWW) viewed from the extracellular side of the membrane. Transport domains in blue, scaffold domains in yellow.

In proteins using the elevator mechanism, the substrate moves some distance across the membrane during the conformational switching. In Table 1, the extent of the movement is indicated as the “vertical distance’, the displacement of the substrate in *z*-direction if the membrane plane is defined as the xy plane. In many cases, the domain movement is more complex than a simple translation, and the total distance over which the substrate is displaced is larger than the vertical distance (Table 1). Structurally, elevator-type membrane transporters show large diversity, indicating that the vertical movement can be realised in multiple ways, but many of the proteins have some characteristics in common. First, the transported substrates bind exclusively, or predominantly, to the transport domain, which is a prerequisite for joined movement of the transport domain and substrate, relative to the rigid scaffold domain. Second, in many cases the transport domain contains structural elements named helical hairpins (HPs) that form the gates, which must be open to allow access of the substrate to the bindings site, and closed to make the elevator movement possible. An open gate prevents sliding of the transport domain relative to the scaffold domain because of steric incompatibility. Third, almost all proteins using elevator transport mechanisms have a membrane topology with inverted repeats [13], resulting in internal pseudosymmetry, which has been used to model the outward-facing conformation based on an inward-facing structure or vice versa [14–17]. Finally, elevator-type transport proteins are often homodimers or homotrimers. Subunit contacts in the oligomers are made exclusively by the scaffold domains, while the transport domains are located peripherally (Figure 3). It is not entirely clear what is the functional significance of the oligomeric state. For homotrimeric members of the glutamate transporter family, it has been shown that the three protomers function independently [18–25], but it is possible that cooperativity may occur in other protein families.

Despite these similarities, global elevator movements and local gating motions vary widely between different protein families (Table 1). Using currently available structural data, elevator mechanisms can be classified into three types with pronounced differences in the way gating is achieved. The classification is based on proteins for which structures are available of multiple conformational states. For many of the proteins in Table 1, only a single structure has been solved, and therefore it is not yet possible to unambiguously classify them.

## Fixed barrier elevator with one gate

The glutamate transporter (SLC1) family of solute transporters is structurally well-characterized with 39 available structures of four different family members: the prokaryotic sodium-dependent aspartate transporters Glt_Ph_ and Glt_Tk_, the human sodium- and potassium-dependent glutamate transporter EAAT1 (Excitatory Amino Acid Transporter 1), and the human neutral amino acid exchanger ASCT2 (Alanine Serine Cysteine Transporter 2) (Table 1 and reviewed in [26]) . While Glt_Ph_ is the prototypical elevator transporter, ASCT2 is the first SLC1 member, for which four key conformations have been resolved structurally: outward-open, outward–occluded [27], inward-open [28] and inward–occluded [29]. We will use these structures to describe the one-gate, fixed barrier elevator movement (Figure 2a).

Like all members of the SLC1 family, neutral amino acid transporter ASCT2 is a homotrimer. Each monomer consists of 8 transmembrane segments (TMs) that form a scaffold domain (TM1–2, TM4–5) and a transport domain (TM3, TM6–8). The transport domain additionally contains two helical hairpins (HP1 and HP2). In the outward-facing states the substrate-binding site is close to the extracellular side of the membrane, and the only difference between open and closed conformations is the position of HP2, which works as a gate to provide access to the binding site from the extracellular aqueous environment [27] (Figure 2a). When the gate is closed, the transported substrate is occluded within the transport domain, which makes the elevator movement possible. The binding site relocates by a distance of ∼19 Å perpendicular to the membrane plane between the outward- to the inward-facing orientation. Strikingly, HP2 was also found to be the gate on the intracellular side, hence the name one-gate elevator mechanism [28]. HP1 plays a role in substrate coordination in the binding site, but in contrast with HP2, it does not change its conformation during the transport cycle. The scaffold domain has two highly tilted helices (TM2 and TM5) along which the transport domain slides. These helices determine the minimal distance that the substrate-binding site must travel, and have been named the fixed barrier [1].

The fixed barrier elevator mechanism with one gate is likely conserved among the SLC1 family, as evidenced by recent single particle cryo-EM structures of Glt_Tk_ [30], and molecular dynamics simulations of Glt_Ph_ [7]. Fixed barrier elevators with one gate may also occur in other families of transporters, for which the number of structurally resolved states is not as large as for the SLC1 family. Transporters of the Phosphotransferase System (PTS), which are responsible for the uptake and phosphorylation of carbohydrates and other compounds such as ascorbate (reviewed in [31]) have characteristic elevator elements, such as transport and scaffold domains, HP gates, and homo-oligomer architecture. Structures of MalT [32,33] and ChbC [34] indicate that they use a fixed barrier and most likely a single gate.

ATP-binding Cassette (ABC) transporters do not use elevator-type mechanisms of transport, with the exception of the non-canonical subfamily of ECF (energy-coupling factor) transporters. ECF transporters are involved in uptake of vitamins or other micronutrients (reviewed in [11]). Two sub-types exist (Group I and II) which may differ in the mechanistic details, but the ensemble of available structural information is consistent with elevator-type behaviour in all ECF transporters. ECF transporters make use of an integral membrane subunit named the S-component that binds the transported substrate on the extracellular side of the membrane (Figure 2d). In many cases, access to the binding site is controlled by two loops, which act as gate (loop 1 and loop 3). In the bound state, with closed gate, the substrate is occluded and the S-component can “topple over” in the membrane, which brings the substrate-binding site to the cytoplasm. In the toppled state the same loops 1 and 3 can move to expose the binding site to the cytoplasm (similar to a one-gate elevator). The S-component may be considered as the equivalent of the transport domain, whereas the counterpart of the scaffold domain is a second integral membrane subunit, named EcfT or T-component (Figure 2d). The use of separate subunits instead of linked domains provides extra functionality, as dissociation and association are part of the transport cycle in some ECF transporters [35]. The EcfT subunit is additionally associated with ATPase subunits for allosteric coupling of the conformational changes to ATP binding and hydrolysis, which are the hallmark of ABC transporters.

## Fixed barrier elevator with two gates

The concentrative nucleoside transporter CNT (a member of the SLC28 family) is a homotrimer [36], with each monomer subdivided into a transport domain (TM1–2, TM4–5, TM7–8 and HP1, HP2) and a scaffold domain (TM3 and TM6). In this case, the binding site for the nucleoside is located at the interface between scaffold and transport domains, but most of the interactions with the substrate come from the residues in the transport domain. CNT uses different gates on the extra- and intracellular sides [36] (Figure 2b). Comparison of structures of CNT in outward-open and outward-closed states revealed different conformations of TM4b, suggesting that this half-TM is an extracellular gate. On the intracellular side, HP1b is the movable element, which gates access to the binding site. The transitions between the outward- and inward-facing states involve a ∼8 Å translocation of the substrate-binding site (perpendicular to the membrane plane), in which it passes a fixed barrier formed by TM3 and TM6 of the scaffold domain. CNT is the only elevator transporter, for which multiple intermediate conformations, where the position of transport domain is distributed between the inward and outward states, have been resolved structurally.

It is possible that the location of the binding site between two domains in CNT necessitates the use of two gates, whereas an occluded binding site within the transport domain, as found in SLC1 transporters, may allow the use of a single gate. Most of the transporters with proposed elevator-like transport mechanisms have substrate-binding sites positioned at the interface of two domains (Table 1). Transporters of AbgT family [37] and the structurally related Na^+^/succinate transporter VcINDY [14] (DASS family), the Na^+^/citrate transporter SeCitS [38] (2HCT family), anion exchanger 1 (AE1), a member of SLC4 family [39] and the structurally related uracil:proton symporter UraA [40,41] from SLC23 family (seven transmembrane segment inverted repeat [42]), and bicarbonate transporter BicA [43] of the SLC26 family are organized in two domains (transport and scaffold) and bind the substrate at the domain interface. All of these proteins may use an elevator mechanism with fixed barrier and two gates [37], but additional structural characterization is needed to classify the gating mechanism of these transporters.

## Moving barrier elevator with two gates

The bile acid transporter ASBT, and structurally related sodium-proton antiporters have 10 and 13 transmembrane helices respectively, with a transport domain (also called core domain) consisting of TM3–5, TM8–10 in ASBT (TM3–5, TM10–12 in sodium-proton antiporters), and a scaffold domain (TM1–2, TM6–7 in ASBT or TM1–2, TM7–9 in sodium-proton antiporters). Despite the movement of the substrate-binding site across the membrane during sliding of the transport domain relative to the scaffold (the hallmark of the elevator mechanism), ASBT does not have a fixed barrier (Figure 2c). Thus, this transporter combines an elevator movement with a moving barrier, which is a typical feature of non-elevator-type mechanisms (Figure 1) [44]. Unlike most other elevator transporters, ASBT and the related sodium-proton antiporters NapA and NhaA do not have helical hairpins. Possibly HPs are suitable for gating when a fixed barrier is used, but are not required for moving barrier elevators (Figure 2c).

ASBT is exceptional among elevator-type transporters because it is a monomeric protein. Another monomeric transporter, for which an elevator mechanism has been postulated, is CcdA [17]. CcdA is the smallest elevator-type protein and is involved in the transport of reducing equivalents from the cytoplasm to the extracellular environment, by using a pair of cysteine residues that can be oxidized to form a disulfide bridge. The protein consists of six transmembrane helices, which are organized in two inverted structural repeats [17]. Comparison of the outward-facing conformation, solved using NMR spectroscopy, and inward-facing conformation, which was computationally modelled using information from the inverted topology, showed that protein forms a unique “O-shaped scaffold” in the centre of which TM1 and TM4 may move as an elevator between inward- and outward-facing states with the active-site cysteines bridging a distance of 12 Å [17]. Structural information on CcdA is still very limited, and further work is required to confirm the elevator mechanism.

## Lipid environment and allosteric inhibition

**Figure 4.**
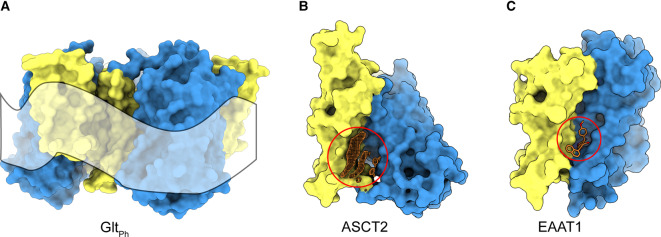
Lipids and elevator transporters. (**a**) deformation of the lipid bilayer around glutamate transporter Glt_Ph_ (PDB ID: 3KBC), when all protomers are in the inward-facing state (adapted from ref. [46]). (**b**) non-protein densities (orange mesh) observed in the neutral amino acid transporter ASCT2 cryo-EM map (EMD-10016) are located at the interface of the transport (blue) and scaffold (yellow) domains and highlighted with a red circle (PDB ID: 6RVX). (**c**) allosteric inhibitor UCPH101 (orange sticks) in excitatory amino acid transporter EAAT1 (PDB ID: 5LLM).

It has been noticed that the TMs of the scaffold domains of many elevator-type transporters are shorter than those in transport domains, and often highly tilted [1]. As a consequence, the distance between the external and internal aqueous solutions is substantially smaller than the thickness of the bulk bilayer. Such thinning not only reduces the extent of elevator movement required to transfer the substrate between the aqueous solutions on either side of the membrane, but may also induce membrane distortion, which in turn could facilitate the sliding movement of the transport domain. Molecular dynamic simulations of ECF transporters in a lipid bilayer predict possible membrane distortion near the EcfT scaffold, which might facilitate toppling of the S-component when it is near the scaffold [11,45]. Recent MD simulations of a lipid bilayer around Glt_Ph_ show different extents of membrane deformation depending on the position of the transport domain [46] (Figure 4a). Protomers of Glt_Ph_ in the outward-facing state induce very little local membrane curvature [46], but the lipid bilayer strongly bends around protomers in the inward-facing state. The energetic penalty of such deformation may be balanced by specific protein–lipid interactions.

Most structures of elevator-type transporters have been determined in the absence of a lipid bilayer, using detergent-solubilized proteins, which precludes accurate analysis of the protein–lipid interface. Nonetheless, these structures can provide indications of specific lipid-binding sites (Figure 4). For example, many non-protein densities were found in structures of ASCT2 determined by single particle cryo-electron microscopy (Figure 4b). These densities likely correspond to phospholipid molecules or cholesterol, although unambiguous identification was not possible at the attained resolution. The observed densities were located around the entire perimeter of the scaffold domain, also in the space between transport and scaffold domains, and close to the substrate binding site [28,29]. Lipids binding at these positions could be important for protein stability and might allosterically affect protein activity. A crystal structure of EAAT1 in the presence of the allosteric inhibitor UCPH101 demonstrated that the inhibitor's binding site is located between transport and scaffold domains [47], exactly where a putative cholesterol molecule was observed in ASCT2 [27–29] (Figure 4c). Also in other families of elevator-type transporters, lipids were found to intercalate between the scaffold and transport domains [38,48]. These observations indicate that specific lipid–protein interactions might affect elevator-like movements of the transporter, and that lipid-binding sites may be targeted for drug design.

In only very few cases have the effects of the lipid environment been studied experimentally. In Glt_Ph_ the relation between lipid composition and transport activity was studied in proteoliposomes. The activity of Glt_Ph_ was higher in liposomes containing the non-bilayer lipid Phosphatidylethanolamine (PE), than in liposomes composed of Phosphatidylcholine (PC) [49]. This effect may be caused by specific interactions between the protein and lipid headgroups, or by colligative properties of the bilayer such as lipid disorder, both of which could affect the elevator-type movements. For ASCT2, glutamine uptake activity in proteoliposomes was enhanced by the presence of cholesterol [29], but again it has not been established whether this effect is due to binding of cholesterol at specific sites, or to colligative effects such as thickness or fluidity. Lipid interactions are also essential for dimer stability of NhaA, which falls apart to monomers in the presence of high detergent concentrations, but is assembled back if cardiolipin is added [50]. *In vivo,* allosteric modulation by lipid molecules has been observed in *Xenopus* oocytes expressing EAAT4 that displayed increased glutamate-induced currents when arachidonic acid was added [51]. The presence of cholesterol was found to be crucial for functioning and localization of EAAT2 [52].

The above examples show that lipids may affect protein function directly via interactions with amino acid residues, which could accelerate or slow down transport domain movements or stabilize the scaffold domain in the membrane. In addition, colligative bilayer properties are likely to affect the functioning of elevator-type transporters, because the lipid–protein interface must rearrange substantially during transport. Finally, also the domain structure of the proteins may affect the bilayer morphology, and consequently elevator dynamics.

## Perspectives

1. **Importance of the field.** Since the first description of an elevator-type transport mechanism for Glt_Ph_ over a decade ago [6], a variety of protein folds have emerged that support elevator movements, not only in secondary active transporters but also in different transporter classes (Table 1). Many of these transporters are potential targets in pharmacological studies and understanding of their transport and gating mechanisms might help with the development of new drugs.
2. **A summary of the current thinking.** In elevator-type transport mechanisms, one protein domain brings the substrate-binding site from one side of the membrane to the other by sliding through the lipid bilayer. The extent of the elevator movement, ranging from 21 Å in Glt_Tk_ to 7.5 Å in ASBT, and number of gating elements (one or two) vary between different proteins (Table 1).
3. **Future directions.** Local deformations of the lipid bilayer near elevator-type transporters, which were observed in MD simulations [46], can be studied experimentally by single particle cryo-electron microscopy, using transporters reconstituted in lipid environment [30], similar to what has been done for the lipid scramblase TMEM16 [53]. Also systematic analysis of the relationship between lipid composition, transport activity and dynamics (for instance by single molecule FRET methods [18,54]) will shed further light on the interplay between bilayer and protein. The gating behaviour might affect the order of binding and release of coupled ions and a substrate, and steady state and pre-steady state kinetic measurements may allow insight in the consequences of using one or two gates [55–61].

## Open Access

Open access for this article was enabled by the participation of University of Groningen in an all-inclusive *Read & Publish* pilot with Portland Press and the Biochemical Society.

## Abbreviations

2HCT: 2-hydroxycarboxylate transporters
ABC: ATP-binding cassette
AbgT: *p*-aminobenzoyl-glutamate transporter
AE1: Anion Exchanger 1
ASBT_NM_: Neisseria meningitidis apical sodium-dependent bile acid transporter
ASBT_Yf_: Yersinia frederiksenii apical sodium-dependent bile acid transporter
ASCT: Alanine Serine Cysteine Transporter
bcChbC: Bacillus cereus chitobiose transporter
bcMalT: Bacillus cereus maltose transporter
BicA: bicarbonate transporter
Bor1: boron exporter 1
CNT_NW_: Neisseria wadworthii concentrative nucleoside transporter
Cryo-EM: cryo-electron microscopy
DASS: divalent anion/Na^+^ symporter
EAAT: Excitatory Amino Acid Transporter
ECF ABC: ECF-type (type III) ABC importers
ECF: Energy Coupling Factor
ECF-FolT: Energy Coupling Factor folate transporter
EcNhaA: Escherichia coli Na^+^/H^+^ antiporter
ecUlaA: Escherichia coli ascorbate transporter (‘utilization of l-ascorbate’)
FRET: Förster Resonance Energy Transfer
Glt_Ph_: Pyrococcus horikoshii glutamate transporter homologue
Glt_Tk_: Thermococcus kadakarensis glutamate transporter homologue
GLUT5: fructose transporter
HP: helical hairpin
KpCitS: Klebsiella pneumonia sodium-ion dependent citrate transporter
LeuT: leucine transporter
LysE: L-lysine exporter
MD: molecular dynamics
MjNhaP1: Methanococcus jannaschii Na^+^/H^+^ antiporter
MtrF: antibiotic exporter (Multiple Transferable Resistance)
NMR: Nuclear Magnetic Resonance
PaNhaP: Pyrococcus abyssi Na^+^/H^+^ antiporter
PC: phosphatidylcholine
PDB: Protein Data Bank
PE: phosphatidylethanolamine
pmUlaA: Pasteurella multocida ascorbate transporter (‘utilization of l-ascorbate’)
PTS: phosphotransferase system
SeCitS: Salmonella enterica sodium-ion dependent citrate transporter
SLC26Dg: *Deinococcus geothermalis* fumarate symporter
TM: transmembrane
TMEM16: lipid scramblase (TransMEMbrane protein)
TtCcdA: *Thermus thermophilus* membrane electron transporter
TtNapA: *Thermus thermophiles* Na^+^/H^+^ antiporter
UapA: purine/H^+^ symporter
UCPH101: 2-amino-4-(4-methoxyphenyl)-7-(naphthalen-1-yl)-5-oxo-5,6,7,8-tetrahydro-4H-chromene-3-carbonitrile
UraA: uracil:proton symporter
vcCNT: *Vibrio cholera* concentrative nucleoside transporter
VcINDY: *Vibrio cholera* Na^+^/succinate transporter (‘I'm not dead yet’)
X-ray: X-ray crystallography
YdaH: antibiotic exporter
SLC: solute carrier
SLC26Dg: Deinococcus geothermalis fumarate symporter
TM: transmembrane
TMEM16: lipid scramblase (TransMEMbrane protein)
TtCcdA: Thermus thermophilus membrane electron transporter
TtNapA: Thermus thermophiles Na^+^/H^+^ antiporter
UapA: purine/H^+^ symporter
UCPH101: 2-amino-4-(4-methoxyphenyl)-7-(naphthalen-1-yl)-5-oxo-5,6,7,8-tetrahydro-4H-chromene-3-carbonitrile
UraA: uracil:proton symporter
vcCNT: Vibrio cholera concentrative nucleoside transporter
VcINDY: Vibrio cholera Na^+^/succinate transporter (‘I'm not dead yet’)
X-ray: X-ray crystallography
YdaH: antibiotic exporter

## References

[1] Drew D. and Boudker O. (2016) Shared molecular mechanisms of membrane transporters. Annu. Rev. Biochem. 85, 543–572 10.1146/annurev-biochem-060815-014520 27023848

[2] Yan N. (2015) Structural biology of the major facilitator superfamily transporters. Annu Rev Biophys 44, 257–283 10.1146/annurev-biophys-060414-033901 26098515

[3] Nomura N., Verdon G., Kang H.J., Shimamura T., Nomura Y., Sonoda Y. et al. (2015) Structure and mechanism of the mammalian fructose transporter GLUT5. Nature 526, 397–401 10.1038/nature14909 26416735 PMC4618315

[4] Yamashita A., Singh S.K., Kawate T., Jin Y. and Gouaux E. (2005) Crystal structure of a bacterial homologue of Na^+^/Cl^–^ dependent neurotransmitter transporters. Nature 437, 215–223 10.1038/nature03978 16041361

[5] Ruprecht J.J., King M.S., Zögg T., Aleksandrova A.A., Pardon E., Crichton P.G. et al. (2019) The molecular mechanism of transport by the mitochondrial ADP/ATP carrier. Cell 176, 435–447.e15 10.1016/j.cell.2018.11.025 30611538 PMC6349463

[6] Reyes N., Ginter C. and Boudker O. (2009) Transport mechanism of a bacterial homologue of glutamate transporters. Nature 462, 880–885 10.1038/nature08616 19924125 PMC2934767

[7] Dechancie J., Shrivastava I.H. and Bahar I. (2011) The mechanism of substrate release by the aspartate transporter Glt Ph: insights from simulations. Mol. Biosyst. 7, 832–842 10.1039/c0mb00175a 21161089 PMC3227142

[8] Bai X., Moraes T.F. and Reithmeier R.A.F. (2017) Structural biology of solute carrier (SLC) membrane transport proteins. Mol. Membr. Biol. 34, 1–32 10.1080/09687688.2018.1448123 29651895

[9] Palmgren M.G. and Nissen P. (2011) P-type ATPases. Annu. Rev. Biophys. 40, 243–266 10.1146/annurev.biophys.093008.131331 21351879

[10] Shi Y. (2013) Common folds and transport mechanisms of secondary active transporters. Annu. Rev. Biophys. 42, 51–72 10.1146/annurev-biophys-083012-130429 23654302

[11] Rempel S., Stanek W.K. and Slotboom D.J. (2019) ECF-Type ATP-binding cassette transporters. Annu. Rev. Biochem. 88, 551–576 10.1146/annurev-biochem-013118-111705 30485755

[12] Rice A.J., Park A. and Pinkett H.W. (2014) Diversity in ABC transporters: type I, II and III importers. Crit. Rev. Biochem. Mol. Biol. 49, 426–437 10.3109/10409238.2014.953626 25155087 PMC4245157

[13] Forrest L.R. (2015) Structural symmetry in membrane proteins. Annu. Rev. Biophys. 44, 311–337 10.1146/annurev-biophys-051013-023008 26098517 PMC5500171

[14] Mulligan C., Fenollar-Ferrer C., Fitzgerald G.A., Vergara-Jaque A., Kaufmann D., Li Y. et al. (2016) The bacterial dicarboxylate transporter VcINDY uses a two-domain elevator-type mechanism. Nat. Struct. Mol. Biol. 23, 256–263 10.1038/nsmb.3166 26828963 PMC5215794

[15] Ficici E., Faraldo-Gómez J.D., Jennings M.L. and Forrest L.R. (2017) Asymmetry of inverted-topology repeats in the AE1 anion exchanger suggests an elevator-like mechanism. J. Gen. Physiol. 149, 1149–1164 10.1085/jgp.201711836 29167180 PMC5715908

[16] Crisman T.J., Qu S., Kanner B.I. and Forrest L.R. (2009) Inward-facing conformation of glutamate transporters as revealed by their inverted-topology structural repeats. Proc Natl Acad Sci 106, 20752–20757 10.1073/pnas.0908570106 19926849 PMC2791632

[17] Zhou Y. and Bushweller J.H. (2018) Solution structure and elevator mechanism of the membrane electron transporter CcdA. Nat. Struct. Mol. Biol. 25, 163–169 10.1038/s41594-018-0022-z 29379172 PMC5805637

[18] Erkens G.B., Hänelt I., Goudsmits J.M.H., Slotboom D.J. and Van Oijen A.M. (2013) Unsynchronised subunit motion in single trimeric sodium-coupled aspartate transporters. Nature 502, 119–123 10.1038/nature12538 24091978

[19] Ruan Y., Miyagi A., Wang X., Chami M., Boudker O. and Scheuring S. (2017) Direct visualization of glutamate transporter elevator mechanism by high-speed AFM. Proc. Natl Acad. Sci. U.S.A. 114, 1584–1588 10.1073/pnas.1616413114 28137870 PMC5320997

[20] Grewer C., Balani P., Weidenfeller C., Bartusel T., Tao Z. and Rauen T. (2005) Individual subunits of the glutamate transporter EAAC1 homotrimer function independently of each other. Biochemistry 44, 11913–11923 10.1021/bi050987n 16128593 PMC2459315

[21] Leary G.P., Stone E.F., Holley D.C. and Kavanaugh M.P. (2007) The glutamate and chloride permeation pathways are colocalized in individual neuronal glutamate transporter subunits. J. Neurosci. 27, 2938–2942 10.1523/JNEUROSCI.4851-06.2007 17360916 PMC6672579

[22] Koch H.P., Brown R.L. and Larsson H.P. (2007) The glutamate-activated anion conductance in excitatory amino acid transporters is gated independently by the individual subunits. J. Neurosci. 27, 2943–2947 10.1523/JNEUROSCI.0118-07.2007 17360917 PMC2435202

[23] Akyuz N., Altman R.B., Blanchard S.C. and Boudker O. (2013) Transport dynamics in a glutamate transporter homologue. Nature 502, 114–118 10.1038/nature12265 23792560 PMC3829612

[24] Stolzenberg S., Khelashvili G. and Weinstein H. (2012) Structural intermediates in a model of the substrate translocation path of the bacterial glutamate transporter homologue GltPh. J. Phys. Chem. B 116, 5372–5383 10.1021/jp301726s 22494242 PMC3350225

[25] Jiang J., Shrivastava I.H., Watts S.D., Bahar I. and Amara S.G. (2011) Large collective motions regulate the functional properties of glutamate transporter trimers. Proc. Natl Acad. Sci. U.S.A. 108, 15141–6 10.1073/pnas.1112216108 21876140 PMC3174670

[26] Arkhipova V., Guskov A. and Slotboom D.J. (2017) Analysis of the quality of crystallographic data and the limitations of structural models. J. Gen. Physiol. 149, 1091–1103 10.1085/jgp.201711852 29089418 PMC5715909

[27] Yu X., Plotnikova O., Bonin P.D., Subashi T.A., McLellan T.J., Dumlao D. et al. (2019) Cryo-EM structures of the human glutamine transporter SLC1A5 (ASCT2) in the outward-facing conformation. eLife 8, 1–17 10.7554/elife.48120 PMC6800002 31580259

[28] Garaeva A.A., Guskov A., Slotboom D.J. and Paulino C. (2019) A one-gate elevator mechanism for the human neutral amino acid transporter ASCT2. Nat. Commun. 10, 1–8 10.1038/s41467-019-11363-x 31366933 PMC6668440

[29] Garaeva A.A., Oostergetel G.T., Gati C., Guskov A., Paulino C. and Slotboom D.J. (2018) Cryo-EM structure of the human neutral amino acid transporter ASCT2. Nat. Struct. Mol. Biol. 25, 515–521 10.1038/s41594-018-0076-y 29872227

[30] Arkhipova V., Guskov A. and Slotboom D.J. (2020) Structural ensemble of a glutamate transporter homologue in lipid nanodisc environment. Nat. Commun. 11, 998 10.1038/s41467-020-14834-8 32081874 PMC7035293

[31] Jeckelmann J.-M. and Erni B. (2019) Carbohydrate transport by group translocation: the bacterial phosphoenolpyruvate: sugar phosphotransferase system. Subcell. Biochem. 92, 223–274 10.1007/978-3-030-18768-2_8 31214989

[32] McCoy J.G., Ren Z., Stanevich V., Lee J., Mitra S., Levin E.J. et al. (2016) The structure of a sugar transporter of the glucose EIIC superfamily provides insight into the elevator mechanism of membrane transport. Structure 24, 956–964 10.1016/j.str.2016.04.003 27161976 PMC4899283

[33] Ren Z., Lee J., Moosa M.M., Nian Y., Hu L., Xu Z. et al. (2018) Structure of an EIIC sugar transporter trapped in an inward-facing conformation. Proc. Natl Acad. Sci. U.S.A. 115, 5962–5967 10.1073/pnas.1800647115 29784777 PMC6003338

[34] Cao Y., Jin X., Levin E.J., Huang H., Zong Y., Quick M. et al. (2011) Crystal structure of a phosphorylation-coupled saccharide transporter. Nature 473, 50–54 10.1038/nature09939 21471968 PMC3201810

[35] Swier L.J.Y.M., Guskov A. and Slotboom D.J. (2016) Structural insight in the toppling mechanism of an energy-coupling factor transporter. Nat. Commun. 7, 11072 10.1038/ncomms11072 27026363 PMC4820897

[36] Hirschi M., Johnson Z.L. and Lee S.Y. (2017) Visualizing multistep elevator-like transitions of a nucleoside transporter. Nature 545, 66–70 10.1038/nature22057 28424521 PMC5567992

[37] Vergara-Jaque A., Fenollar-Ferrer C., Mulligan C., Mindell J.A. and Forrest L.R. (2015) Family resemblances: A common fold for some dimeric ion-coupled secondary transporters. J. Gen. Physiol. 146, 423–434 10.1085/jgp.201511481 26503722 PMC4621753

[38] Wöhlert D., Grötzinger M.J., Kühlbrandt W. and Yildiz Ö (2015) Mechanism of Na^+^-dependent citrate transport from the structure of an asymmetrical CitS dimer. eLife 4, 1–18 10.7554/eLife.09375 PMC4718727 26636752

[39] Arakawa T., Kobayashi-Yurugi T., Alguel Y., Iwanari H., Hatae H., Iwata M. et al. (2015) Crystal structure of the anion exchanger domain of human erythrocyte band 3. Science 350, 680–684 10.1126/science.aaa4335 26542571

[40] Yu X., Yang G., Yan C., Baylon J.L., Jiang J., Fan H. et al. (2017) Dimeric structure of the uracil:proton symporter UraA provides mechanistic insights into the SLC4/23/26 transporters. Cell Res. 27, 1020–1033 10.1038/cr.2017.83 28621327 PMC5539350

[41] Lu F., Li S., Jiang Y., Jiang J., Fan H., Lu G. et al. (2011) Structure and mechanism of the uracil transporter UraA. Nature 472, 243–247 10.1038/nature09885 21423164

[42] Chang Y.N. and Geertsma E.R. (2017) The novel class of seven transmembrane segment inverted repeat carriers. Biol. Chem. 398, 165–174 10.1515/hsz-2016-0254 27865089

[43] Wang C., Sun B., Zhang X., Huang X., Zhang M., Guo H. et al. (2019) Structural mechanism of the active bicarbonate transporter from cyanobacteria. Nat. Plants 5, 1184–1193 10.1038/s41477-019-0538-1 31712753

[44] Zhou X., Levin E.J., Pan Y., McCoy J.G., Sharma R., Kloss B. et al. (2014) Structural basis of the alternating-access mechanism in a bile acid transporter. Nature 505, 569–573 10.1038/nature12811 24317697 PMC4142352

[45] Faustino, I., Abdizadeh H., Souza P.C.T., Jeucken A., Stanek W.K., Guskov A. et al. (2020) Membrane mediated toppling mechanism of the folate energy coupling factor transporter. Nat. Commun. 11, 1763 10.1038/s41467-020-15554-9 32273501 PMC7145868

[46] Zhou W., Fiorin G., Anselmi C., Karimi-Varzaneh H.A., Poblete H., Forrest L.R. et al. (2019) Large-scale state-dependent membrane remodeling by a transporter protein. eLife 8, 1–32 10.7554/eLife.50576 PMC6957315 31855177

[47] Canul-Tec J.C., Assal R., Cirri E., Legrand P., Brier S., Chamot-Rooke J. et al. (2017) Structure and allosteric inhibition of excitatory amino acid transporter 1. Nature 544, 446–451 10.1038/nature22064 28424515 PMC5410168

[48] Coincon M., Uzdavinys P., Nji E., Dotson D.L., Winkelmann I., Abdul-Hussein S. et al. (2016) Crystal structures reveal the molecular basis of ion translocation in sodium/proton antiporters. Nat. Struct. Mol. Biol. 23, 248–255 10.1038/nsmb.3164 26828964

[49] McIlwain B.C., Vandenberg R.J. and Ryan R.M. (2015) Transport rates of a glutamate transporter homologue are influenced by the lipid bilayer. J. Biol. Chem. 290, 9780–9788 10.1074/jbc.M114.630590 25713135 PMC4392276

[50] Rimon A., Mondal R., Friedler A. and Padan E. (2019) Cardiolipin is an optimal phospholipid for the assembly, stability, and proper functionality of the dimeric form of NhaA Na^+^/H^+^ antiporter. Sci. Rep. 9, 1–11 10.1038/s41598-019-54198-8 31776461 PMC6881326

[51] Fairman W.A., Sonders M.S., Murdoch G.H. and Amara S.G. (1998) Arachidonic acid elicits a substrategated proton current associated with the glutamate transporter EAAT4. Nat. Neurosci. 1, 105–113 10.1038/355 10195124

[52] Butchbach M.E.R., Tian G., Guo H. and Lin C.L.G. (2004) Association of excitatory amino acid transporters, especially EAAT2, with cholesterol-rich lipid raft microdomains: Importance for excitatory amino acid transporter localization and function. J. Biol. Chem. 279, 34388–34396 10.1074/jbc.M403938200 15187084

[53] Kalienkova V., Mosina V.C., Bryner L., Oostergetel G.T., Dutzler R. and Paulino C. (2019) Stepwise activation mechanism of the scramblase nhtmem16 revealed by cryo- em. eLife 8, 1–27 10.7554/eLife.44364 PMC6414200 30785398

[54] Akyuz N., Georgieva E.R., Zhou Z., Stolzenberg S., Cuendet M.A., Khelashvili G. et al. (2015) Transport domain unlocking sets the uptake rate of an aspartate transporter. Nature 518, 68–73 10.1038/nature14158 25652997 PMC4351760

[55] Ewers D., Becher T., Machtens J.P., Weyand I. and Fahlke C. (2013) Induced fit substrate binding to an archeal glutamate transporter homologue. Proc. Natl Acad. Sci. U.S.A. 110, 12486–12491 10.1073/pnas.1300772110 23840066 PMC3725095

[56] Zhang Z., Tao Z., Gameiro A., Barcelona S., Braams S., Rauen T. et al. (2007) Transport direction determines the kinetics of substrate transport by the glutamate transporter EAAC1. Proc. Natl Acad. Sci. U.S.A. 104, 18025–18030 10.1073/pnas.0704570104 17991780 PMC2084290

[57] Hänelt I., Jensen S., Wunnicke D. and Slotboom D.J. (2015) Low affinity and slow Na^+^ binding precedes high affinity aspartate binding in the secondary-active transporter gltPh. J. Biol. Chem. 290, 15962–15972 10.1074/jbc.M115.656876 25922069 PMC4481202

[58] Oh S. and Boudker O. (2018) Kinetic mechanism of coupled binding in sodium-aspartate symporter GltPh. Elife 7, 1–20 10.7554/eLife.37291 PMC6175574 30255846

[59] Ravera S., Quick M., Nicola J.P., Carrasco N. and Mario Amzel L. (2015) Beyond non-integer Hill coefficients: a novel approach to analyzing binding data, applied to Na^+^-driven transporters. J Gen Physiol 145, 555–563 10.1085/jgp.201511365 26009546 PMC4442788

[60] Lolkema J.S. and Slotboom D.J. (2019) Models to determine the kinetic mechanisms of ioncoupled transporters. J. Gen. Physiol. 151, 369–380 10.1085/jgp.201812055 30630873 PMC6400521

[61] Burtscher V., Schicker K., Freissmuth M. and Sandtner W. (2019) Kinetic models of secondary active transporters. Int. J. Mol. Sci. 20, E5365 10.3390/ijms20215365 31661895 PMC6862442

[62] Jensen S., Guskov A., Rempel S., Hänelt I. and Slotboom D.J. (2013) Crystal structure of a substrate-free aspartate transporter. Nat. Struct. Mol. Biol. 20, 1224–1226 10.1038/nsmb.2663 24013209

[63] Guskov A., Jensen S., Faustino I., Marrink S.J. and Slotboom D.J. (2016) Coupled binding mechanism of three sodium ions and aspartate in the glutamate transporter homologue gltTk. Nat. Commun. 7, 13420 10.1038/ncomms13420 27830699 PMC5110648

[64] Arkhipova V., Trinco G., Ettema T.W., Jensen S., Slotboom D.J. and Guskov A. (2019) Binding and transport of D-aspartate by the glutamate transporter homolog Glt Tk. eLife 8, 1–12 10.7554/eLife.45286 PMC6482001 30969168

[65] Yernool D., Boudker O., Jin Y. and Gouaux E. (2004) Structure of a glutamate transporter homologue from *Pyrococcus horikoshii*. Nature 431, 811–818 10.1038/nature03018 15483603

[66] Boudker O., Ryan R.M., Yernool D., Shimamoto K. and Gouaux E. (2007) Coupling substrate and ion binding to extracellular gate of a sodium-dependent aspartate transporter. Nature 445, 387–393 10.1038/nature05455 17230192

[67] Reyes N., Oh S. and Boudker O. (2013) Binding thermodynamics of a glutamate transporter homolog. Nat. Struct. Mol. Biol. 20, 634–640 10.1038/nsmb.2548 23563139 PMC3711778

[68] Verdon G., Oh S.C., Serio R. and Boudker O. (2014) Coupled ion binding and structural transitions along the transport cycle of glutamate transporters. eLife 2014, 1–23 10.7554/eLife.02283 PMC4051121 24842876

[69] Scopelliti A.J., Font J., Vandenberg R.J., Boudker O. and Ryan R.M. (2018) Structural characterisation reveals insights into substrate recognition by the glutamine transporter ASCT2/SLC1A5. Nat. Commun. 9, 1–12 10.1038/s41467-017-02444-w 29295993 PMC5750217

[70] Verdon G. and Boudker O. (2012) Crystal structure of an asymmetric trimer of a bacterial glutamate transporter homolog. Nat. Struct. Mol. Biol. 19, 355–357 10.1038/nsmb.2233 22343718 PMC3633560

[71] Johnson Z.L., Cheong C.G. and Lee S.Y. (2012) Crystal structure of a concentrative nucleoside transporter from *Vibrio cholerae* at 2.4 Å. Nature 483, 489–493 10.1038/nature10882 22407322 PMC3310960

[72] Johnson Z.L., Lee J.H., Lee K., Lee M., Kwon D.Y., Hong J. et al. (2014) Structural basis of nucleoside and nucleoside drug selectivity by concentrative nucleoside transporters. eLife 3, 1–19 10.7554/eLife.03604 PMC4139061 25082345

[73] Hu N.J., Iwata S., Cameron A.D. and Drew D. (2011) Crystal structure of a bacterial homologue of the bile acid sodium symporter ASBT. Nature 478, 408–411 10.1038/nature10450 21976025 PMC3198845

[74] Thurtle-Schmidt B.H. and Stroud R.M. (2016) Structure of Bor1 supports an elevator transport mechanism for SLC4 anion exchangers. Proc. Natl Acad. Sci. U.S.A. 113, 10542–10546 10.1073/pnas.1612603113 27601653 PMC5035872

[75] Coudray N., Seyler S L., Lasala R., Zhang Z., Clark K.M., Dumont M.E. et al. (2017) Structure of the SLC4 transporter Bor1p in an inward-facing conformation. Protein Sci. 26, 130–145 10.1002/pro.3061 27717063 PMC5192975

[76] Alguel Y., Amillis S., Leung J., Lambrinidis G., Capaldi S., Scull N.J. et al. (2016) Structure of eukaryotic purine/H^+^ symporter UapA suggests a role for homodimerization in transport activity. Nat. Commun. 7, 1–9 10.1038/ncomms11336 PMC4837479 27088252

[77] Geertsma E.R., Chang Y.N., Shaik F.R., Neldner Y., Pardon E., Steyaert J. et al. (2015) Structure of a prokaryotic fumarate transporter reveals the architecture of the SLC26 family. Nat. Struct. Mol. Biol. 22, 803–808 10.1038/nsmb.3091 26367249

[78] Su C.C., Bolla J.R., Kumar N., Radhakrishnan A., Long F., Delmar J.A. et al. (2015) Structure and function of *Neisseria gonorrhoeae* MtrF illuminates a class of antimetabolite efflux pumps. Cell Rep. 11, 61–70 10.1016/j.celrep.2015.03.003 25818299 PMC4410016

[79] Bolla J.R., Su C.C., Delmar J.A., Radhakrishnan A., Kumar N., Chou T.H. et al. (2015) Crystal structure of the *Alcanivorax borkumensis* YdaH transporter reveals an unusual topology. Nat. Commun. 6, 1–10 10.1038/ncomms7874 PMC4410182 25892120

[80] Kim J.W., Kim S., Kim S., Lee H., Lee J.O. and Jin M.S. (2017) Structural insights into the elevator-like mechanism of the sodium/citrate symporter CitS. Sci. Rep. 7, 1–10 10.1038/s41598-017-02794-x 28566738 PMC5451387

[81] Kebbel F., Kurz M., Arheit M., Grütter M.G. and Stahlberg H. (2013) Structure and substrate-induced conformational changes of the secondary citrate/sodium symporter CitS revealed by electron crystallography. Structure 21, 1243–1250 10.1016/j.str.2013.05.011 23810698

[82] Mancusso R., Gregorio G.G., Liu Q. and Wang D.N. (2012) Structure and mechanism of a bacterial sodium-dependent dicarboxylate transporter. Nature 491, 622–626 10.1038/nature11542 23086149 PMC3617922

[83] Hunte C., Screpanti E., Venturi M., Rimon A., Padan E. and Michel H. (2005) Structure of a Na^+^/H^+^ antiporter and insights into mechanism of action and regulation by pH. Nature 435, 1197–1202 10.1038/nature03692 15988517

[84] Lee C., Yashiro S., Dotson D.L., Uzdavinys P., Iwata S., Sansom M.S.P. et al. (2014) Crystal structure of the sodium-proton antiporter NhaA dimer and new mechanistic insights. J. Gen. Physiol. 144, 529–544 10.1085/jgp.201411219 25422503 PMC4242812

[85] Appel M., Hizlan D., Vinothkumar K.R., Ziegler C. and Kühlbrandt W. (2009) Conformations of NhaA, the Na/H exchanger from *Escherichia coli*, in the pH-activated and ion-translocating states. J. Mol. Biol. 386, 351–365 10.1016/j.jmb.2008.12.042 19135453

[86] Lee C., Kang H.J., Von Ballmoos C., Newstead S., Uzdavinys P., Dotson D.L. et al. (2013) A two-domain elevator mechanism for sodium/proton antiport. Nature 501, 573–577 10.1038/nature12484 23995679 PMC3914025

[87] Paulino C., Wöhlert D., Kapotova E., Yildiz Ö. and Kühlbrandt W. (2014) Structure and transport mechanism of the sodium/proton antiporter MjNhaP1. eLife 3, 1–21 10.7554/eLife.03583 PMC4381896 25426803

[88] Wöhlert D., Kühlbrandt W. and Yildiz O. (2014) Structure and substrate ion binding in the sodium/proton antiporter PaNhaP. eLife 3, e03579 10.7554/eLife.03579 25426802 PMC4381880

[89] Luo P., Yu X., Wang W., Fan S., Li X. and Wang J. (2015) Crystal structure of a phosphorylation-coupled vitamin C transporter. Nat. Struct. Mol. Biol. 22, 238–241 10.1038/nsmb.2975 25686089

[90] Luo P., Dai S., Zeng J., Duan J., Shi H. and Wang J. (2018) Inward-facing conformation of l-ascorbate transporter suggests an elevator mechanism. Cell Discov. 4, 1–9 10.1038/s41421-018-0037-y 30038796 PMC6048161

[91] Yu Y., Zhou M., Kirsch F., Xu C., Zhang L., Wang Y. et al. (2014) Planar substrate-binding site dictates the specificity of ECF-type nickel/cobalt transporters. Cell Res. 24, 267–277 10.1038/cr.2013.172 24366337 PMC3945884

[92] Bao Z., Qi X., Hong S., Xu K., He F., Zhang M. et al. (2017) Structure and mechanism of a group-I cobalt energy coupling factor transporter. Cell Res. 27, 675–687 10.1038/cr.2017.38 28322252 PMC5520853

[93] Zhang P., Wang J. and Shi Y. (2010) Structure and mechanism of the S component of a bacterial ECF transporter. Nature 468, 717–720 10.1038/nature09488 20972419

[94] Erkens G.B., Berntsson R.P.A., Fulyani F., Majsnerowska M., Vujiĉić-Žagar A., Ter Beek J. et al. (2011) The structural basis of modularity in ECF-type ABC transporters. Nat. Struct. Mol. Biol. 18, 755–760 10.1038/nsmb.2073 21706007

[95] Berntsson R.P.A., Ter Beek J., Majsnerowska M., Duurkens R.H., Puri P., Poolman B. et al. (2012) Structural divergence of paralogous S components from ECF-type ABC transporters. Proc. Natl Acad. Sci. U.S.A. 109, 13990–13995 10.1073/pnas.1203219109 22891302 PMC3435211

[96] Karpowich N.K., Song J. and Wang D.N. (2016) An aromatic Cap seals the substrate binding site in an ECF-type S subunit for riboflavin. J. Mol. Biol. 428, 3118–3130 10.1016/j.jmb.2016.06.003 27312125 PMC4975955

[97] Swier L.J.Y.M., Monjas L., Guskov A., De Voogd A.R., Erkens G.B., Slotboom D.J. et al. (2015) Structure-based design of potent small-molecule binders to the S-component of the ECF transporter for thiamine. ChemBioChem 16, 819–826 10.1002/cbic.201402673 25676607

[98] Zhao Q., Wang C., Wang C., Guo H., Bao Z., Zhang M. et al. (2015) Structures of FolT in substrate-bound and substrate-released conformations reveal a gating mechanism for ECF transporters. Nat. Commun. 6, 1–7 10.1038/ncomms8661 PMC4525288 26198469

[99] Rempel S., Colucci E., de Gier J.W., Guskov A. and Slotboom D.J. (2018) Cysteine-mediated decyanation of vitamin B12 by the predicted membrane transporter BtuM. Nat. Commun. 9, 1–8 10.1038/s41467-018-05441-9 30072686 PMC6072759

[100] Santos J.A., Rempel S., Mous S.T.M., Pereira C.T., Ter B.J., de Gier J.W. et al. (2018) Functional and structural characterization of an ECF-type ABC transporter for vitamin B12. eLife 7, 1–16 10.7554/eLife.35828 PMC5997447 29809140

[101] Zhang M., Bao Z., Zhao Q., Guo H., Xu K., Wang C. et al. (2014) Structure of a pantothenate transporter and implications for ECF module sharing and energy coupling of group II ECF transporters. Proc. Natl Acad. Sci. U.S.A. 111, 18560–18565 10.1073/pnas.1412246112 25512487 PMC4284524

[102] Xu K., Zhang M., Zhao Q., Yu F., Guo H., Wang C. et al. (2013) Crystal structure of a folate energy-coupling factor transporter from lactobacillus brevis. Nature 497, 268–271 10.1038/nature12046 23584589

[103] Wang T., Fu G., Pan X., Wu J., Gong X., Wang J. et al. (2013) Structure of a bacterial energy-coupling factor transporter. Nature 497, 272–276 10.1038/nature12045 23584587

